# “How can our children learn from us about our way of life or understand who they are?”: Residential schools and their impact on the wellbeing of Indigenous youth in Attapadi, South India

**DOI:** 10.1177/13634615231213834

**Published:** 2024-02-25

**Authors:** Mathew Sunil George, K. A. Ramu, Rajendra Prasad, N. S. Prashanth, Susheela Kenjoor, Janie Busby Grant

**Affiliations:** 1 Health Research Institute, 2234 University of Canberra, Canberra, ACT, Australia; 2 Karrara, Attapadi, Palakkad, Kerala, India; 3 THAMPU, Sastha Temple Road, Kochi, Kerala, India; 4 Institute of Public Health, Banashankari Stage II, Bengaluru, Karnataka, India; 5 Department of Social Work, Mysore University, Mysuru, Karnataka, India; 6 Faculty of Health, 2234 University of Canberra, Canberra, ACT, Australia

**Keywords:** Adivasi, India, Indigenous, mental health, residential schools

## Abstract

Residential schools are commonly used in India to provide education for Indigenous youth, which requires young people to stay for long periods at distance from their families and communities. Internationally, there is clear evidence for the deleterious effects of residential schools on the mental health and social and community outcomes of Indigenous children, however little is known about the Indian Indigenous experience. This study examined the impact of residential schooling on Indigenous children's wellbeing and that of their communities, using data from an ethnographic research project in Attapadi, Kerala, including interviews, focus group discussions and participant observation with Indigenous communities. Key outcomes from residential schooling reported by the participants include the fear of losing Indigenous identity, shame of being Indigenous, change in the attitude of young people when they returned from schools, and feelings of confusion and stress that young Indigenous participants felt trying to fit into their communities on their return. Findings suggest that these Indigenous youth felt disconnected from several factors that are known to promote resilience for Indigenous communities including a strong cultural identity, connection to the land and ancestors, thereby making them more vulnerable to poor mental health and negative impacts on their overall wellbeing. Addressing these concerns requires a detailed understanding of the specific factors influencing outcomes for Indigenous youth within the Indian residential schooling system, and designing and implementing data-informed conceptual, structural and policy change including the provision of culturally safe mental health services.

## Introduction

There is a growing body of evidence on the crucial role played by culture in the wellbeing of Indigenous communities across the world. Culture informs and permeates every aspect of Indigenous life including the understanding of the self, interpersonal relationships, and relationships with the environment (Grant, 2004). A strong cultural identity underpins the social and emotional wellbeing of Indigenous peoples (Social Health Reference Group, 2004), promoting resilience, enriching self-esteem, and reducing vulnerability to mental health stressors (Dockery, 2010; Fleming & Ledogar, 2008; Houkamau & Sibley, 2011). Enculturation, the process by which Indigenous communities learn about and identify with their traditional ethnic culture and affirmation of one's heritage, has shown to be a protective factor reducing the risk of maladaptive outcomes including substance abuse and stress (Yoder et al., 2006).

Two key mechanisms through which young Indigenous people learn about their identity, heritage and unique social norms relevant to their community are oral tradition and experiential learning. Oral traditions, the transmission of aspects of culture, identity and social codes in the form of stories from one generation to the next, is an important means of sustaining Indigenous identity and traditions (Mahuika, 2019). For Indigenous communities across the world, this includes creation stories, connections to the land, historical accounts, and language. A second mechanism critical to the development of Indigenous identities is the process of experiential learning. This involves, but is not limited to, learning from the land, elders, traditions and ceremonies, community, wider familial and parental support systems. Through this process, young Indigenous people in particular use their direct experiences to understand what it means to be an Indigenous person in their particular community (Kaminski, 2012). Young Indigenous people who grow up embedded in their communities learn from their elders and from their experiences on country about who they are through their unique cultural and social codes, thereby leading to the evolution of their self-identity.

Historically, colonisation and its associated institutions and their impacts were key mechanisms that disrupted the cultural identities of Indigenous communities. The forcible occupation of the traditional lands of Indigenous communities by colonial powers was closely followed by Indigenous communities being forced to assimilate by rejecting their cultures, identities, and values in favour of that of the colonisers. Assimilation has been shown to be associated with psychological stress (Berry, 1970, 1997).

One key strategy of assimilation used in many of these colonial contexts was (and in some countries, continues to be) the practice of taking Indigenous children from their families and communities and placing them in residential schools (Smith, 2009; Wilk et al., 2017). Typically, these schools were located away from Indigenous communities and imbued with specific features that operationalised the explicit state policy of eradicating Indigenous identity and culture from children and replacing them with the values and knowledge of dominant coloniser communities (Adams, 1995; Human Rights and Equal Opportunity Commission, 1997; Trafzer et al., 2006). The curriculum typically reflected the values and life experiences of non-Indigenous communities. Indigenous characters and examples when present in such curricula were usually presented as secondary or lower to non-Indigenous examples (Kundu, 1994; Sundar, 2010). Hardly any contact with family was allowed, the day was regimented with strict timetables, and any deviations could be punished with physical, emotional and verbal abuse (Marshall & Gallant, 2021; Milloy, 2017; Smith, 2009; Truth and Reconciliation Commission of Canada, 2015a). Integration into “society”, which in reality meant adopting the values and ways of life of the dominant non-Indigenous communities, was assumed to be the only way to ensure that Indigenous communities had a future, participated in mainstream economic activities and improved their standard of living (Davin, 1879; Smith, 2009; Sommerlad, 1976). Students were typically forbidden to speak their own languages at these schools, and their cultures and heritages were debased (Jung, 2009; Logan, 2016). Reports studying residential schools for Indigenous communities in Canada have stated that the explicit purpose of the residential schools was to assimilate Aboriginal children and instil a sense of shame regarding their culture so that they no longer existed as distinct peoples (Bombay et al., 2014; Marshall & Gallant, 2021; Truth and Reconciliation Commission of Canada, 2015b).

In recent years, extensive research has established the outcomes of residential school policies on the health and wellbeing of Indigenous peoples across the world. The reports of the Truth and Reconciliation Commission in Canada, and the Stolen Generations in Australia, both of which investigated the practice of forced removal of children of Indigenous communities in their respective countries, established beyond doubt the harm these practices have caused (Human Rights and Equal Opportunity Commission, 1997; Social Health Reference Group, 2004). While these policies were implemented with the “paternalistic” intention of providing better education and through it various opportunities later in life for children from Indigenous communities, deprivation due to separation from parents, community, country and culture led to severe trauma, psychological distress and injury, the effects of which are still being felt today (Menzies, 2010; Royal Australian & New Zealand College of Psychiatrists, 2020). In addition to the negative impacts on health and wellbeing, recent evidence also shows that intergenerational trauma among the Indigenous communities has led to a range of long-term adverse cultural and socioeconomic outcomes (Australian Institute of Health and Welfare, 2018; Menzies, 2010; O’Neill et al., 2018).

## Approach to the education of Indigenous young people in India

Indigenous^1^ communities, officially delineated “Scheduled Tribes”, constitute 8% of the Indian population and are found in every state in India (Office of the Registrar General & Census Commissioner, 2011). The place of Indigenous communities in modern Indian society has been constructed on feudal, colonial, and imperialistic notions that combine traditional and historical concepts of notions of progress and development as determined by dominant communities who more or less control the political processes. Prior to India's Independence, sporadic efforts were carried out to provide formal education to Indigenous communities in India, primarily by Christian missionaries (Xaxa, 2011). The first Indian residential school for Indigenous communities along the lines of the “Ashrama Shala”^2^ was set up in 1923 for children from the Bhil community in the state of Gujarat. Similar schools were set up in Odisha in 1939. The stated objective of these residential schools was to encourage Indigenous children to take up schooling and improve their social status (B. C. Mishra & Dhir, 2005). At the national level, the first steps to address the issue of education of Indigenous communities in India took place post-independence with the establishment of the Dhebar Commission in 1960 (Xaxa et al., 2014). The Dhebar Commission proposed the “Adivasi Ashram Shala” scheme as the path to improve the educational status of Indigenous young people in India. Since then, Ashram Shalas (residential schools) have been a key part of the Tribal Sub-Plan^3^ during the five-year plans.

Despite the view of the first Prime Minister that education for the Indigenous was not meant to assimilate them or train them to carry out tasks for the administration, over the years, Indian educational policy has explicitly followed an assimilation approach (Xaxa et al., 2014). Assimilation, which is a key strategy in the process of colonisation, was used to discredit and disrupt Indigenous cultures and established the superiority and dominance of upper caste forms of knowledge and the transmission of knowledge. This colonial approach and the inherent structural racism perpetuated by the caste system in India (in which Indigenous communities are considered to be at the bottom of the social hierarchy) led to the privileging of the Ashrama Shala template for the education of Indigenous young people in India in the form of residential schools. In this process of assimilation the boarding (residential) school, which Indigenous children are required to attend from a young age (5–8 years), is a key first step. As seen in similar past programs in other countries, most of these schools, whether administered by the government or through private foundations, are located away from Indigenous villages and require their students to learn and converse only in the official language of the state (Gupta & Padel, 2020). The curriculum adopted at these schools is designed around the experiences of non-Indigenous communities and has few connections to the culture, history or issues of the Indigenous people (Kundu, 1994). Textbooks which are used as the key mode of transmitting cultural messages in these schools tend to have Indigenous characters as “backward”, “strange” or exotic (Kumar, 1989; Kundu, 1994). Regimented timetables, a focus on competitive assessments through exams, and learning that is not connected to the work process are all features of these schools that are alien to the culture and learning styles of Indigenous communities (Sundar, 2010). These features of residential schools, which are foreign to the culture of Indigenous communities, are examples of the structural racism that is implicit in the current educational system. Children remain at these residential schools from approximately 5 to 17 years of age and are generally permitted to return home only once or twice per year. Reports about Indigenous children living in residential schools in India have raised concerns similar to those that have been witnessed elsewhere in the world. These include physical and verbal abuse, poor infrastructure, overcrowding, suicides, and deaths of children studying at these schools (Bal Hakka Abhiyan, 2017; Nambisan, 2000; Sharma, 2016). At the policy level, the concurrent listing of education (allowing for policy making at both the federal and state levels) has also led to the mushrooming of various schemes for Indigenous education with diverse structures governing Indigenous education in different states of India (Veerbhadranaika et al., 2012).

The impact of the residential school system on Indigenous Indian youth has not yet been established, but based on outcomes in other countries is likely to be multifactorial and associated with significant negative impacts on health and wellbeing. Physical separation from their communities and families is likely to result in the loss of opportunities to learn from their elders and country about their unique identity and cultural norms via oral tradition and experiential learning. Given that language plays a key role in connecting a person to their ethnic group and helps shape a person's identity (Standing Committee on Aboriginal and Torres Strait Islander Affairs, 2012), marginalisation of Indigenous languages under the guise of modernisation and education prevents Indigenous youth from learning key concepts from their communities. In the long term this is likely to lead to the destruction of Indigenous cultures (Abbi, 2012). Further, official attempts to ensure that Indigenous children assimilate the dominant community values and norms furthers the process of alienation of young Indigenous people from their communities (Nikitin et al., 2010; Stiller et al., 2020). Based on data from other contexts, this process is likely to engender substantial psychological distress among Indigenous youth and follow them throughout their lives. On various physical health and economic indicators regarding life expectancy, maternal and child health, enrolment in schools and academic performance as well as access to services, Indigenous communities in India are substantially behind other social groups in India (Das et al., 2012; Ministry of Health and Family Welfare/Ministry of Tribal Affairs, 2018; Stiller et al., 2020). There is also a substantial difference (14 percentage point gap) between the literacy rate of Indigenous communities and others in India. School dropout rates between grades 1 to 9 is around 70%, which is 20% higher than the national average for other communities (Baxi & Prasad, 2019).

## Education of Indigenous young people in Attapadi

The approach to the education of Indigenous children in Attapadi follows the same pattern implemented across India. Indigenous students are taught primarily in government institutions (see Table 1). A few private schools also exist in Attapadi and cater to the education of a small proportion of Indigenous children.

**Table 1.** Public School arrangements for Indigenous children from Attapadi

Level of schooling	No of institutions	Age range (yrs.)	Classes	Location	Residential
Anganwadi^a^ (Play school)	175	3-5	Pre-school	In or near the village	No
Lower Primary School	20	5-10	I-IV	In each panchayat^b^	Primarily home but some in hostel
Upper Primary School	10	10-13	V-VII	At the block panchayat level	Mix of home and hostel
High School	8	14-17	VIII-X	Block panchayat	Primarily hostel
Higher^c^ Secondary	6	18-19	XI-XII	Outside Attapadi	Primarily hostel

^a^ An anganwadi is type of rural childcare centre in India and is part of the Integrated Child Development Services program. An anganwadi provides non-formal pre-school education and a range of other services including supplementary nutrition, immunisation, health check-ups and health education.

^b^ A panchayat is an elected village level council in India

^c^ Two Vocational Training schools that train Indigenous children in various trades are also functional in Attapadi for those children who complete high school and choose not to enrol for higher secondary. These schools do not have any hostels attached to them.

Even when schools are located in Attapadi, students whose homes are in remote villages and those identified as falling behind in their studies are required to stay in the hostel and attend classes. If school or hostels do not have the capacity to accommodate all students, then some are sent to schools outside Attapadi where there are vacancies in hostels. Even when students are staying in hostel located in Attapadi, they are not permitted to go home to meet their families except during school holidays.

The effects of residentials schools on Indigenous children and their communities emerged as a salient issue among broader discussions about health and psychological wellbeing among Indigenous community members living in Attapadi during research conducted by the first author. The purpose of this article is to gain an understanding of the effects of residentials schooling practices on the wellbeing of Indigenous children and their communities.

## Methods

### Study setting

Attapadi is located in the Palakkad district in the south Indian state of Kerala and is home to the Muduga, Kurumba and Irula Indigenous communities who live in 192 villages. Attapadi is primarily a hilly area and is bordered on the east by Coimbatore district, Tamil Nadu, on the north by the Nilgiris and on the south by Mannarghat and Ernad taluks and on the west by Karimba village. Bhavani and Siruvani are the two main rivers flowing through Attapadi and the Silent Valley National Park is located here. As per the last census data, Indigenous communities in Attapadi comprised around 44% of the population and consists of 26,908 Irulas, 2,551 Mudugas and 3,497 Kurumbas respectively (Registrar General of India, 2011).

### Data collection

The data used in this article is a subset of research from a larger study that focused on inequity in access to healthcare for these communities, which adopted ethnographic fieldwork with Indigenous stakeholders to identify structural racism, power differentials and a lack of culturally respectful care as barriers to healthcare (George et al., 2020). Fieldwork was conducted between August 2018 and January 2019 and again between August 2019 and October 2019 by the first author who is fluent in the native languages of the participants and had prior experience of working with marginalised communities in India. RKA, the second author, assisted the first author during fieldwork and organising visits to the fieldwork sites (villages). During fieldwork all traditional protocols that govern visits by non-Indigenous individuals among those Indigenous communities were followed. In these communities this first entailed discussions with the village chief, who functions as the designated gatekeeper and spiritual authority for that community. After these initial meetings, which focused on study purpose and methods, the village chief then consulted with the village council to decide on provision of entry and interaction rights for the researcher. Once this permission had been granted the researcher could then begin discussions with individuals within the community regarding the study, obtaining informed consent from any individuals who were interested in participating in any aspect of the study.

In-depth interviews (IDIs), focus group discussions (FGDs) and participant observations (PO) were utilised to collect ethnographic data from the various stakeholders who took part in this study. Among community participants, IDIs took place at their homes, whereas FGDs took place in the common courtyard in the villages. Both men and women from the villages participated in the FGDs as per the traditional customs of the communities where issues of common interest were discussed by everyone with the village chief presiding over it. Among health system participants and key informants, IDIs took place at a location that they preferred. IDIs and FGDs were audio-recorded, transcribed, translated into English and cross-checked against the original recordings to verify the accuracy. PO were carried out in the villages and helped document the dynamics of interactions between the older community members and youth as well Indigenous customs, health traditions and related cultural practices.

### Sampling

Theoretical sampling, with initial interviews providing new topics that were explored in subsequent interviews, was used to identify participants (Charmaz, 2006). Thus, when the practice of children studying in residential schools was identified as a determinant of exclusion during initial interviews, this was added to the topic guide and explored with participants in subsequent interviews. Forty-one IDIs and six FGDs were conducted with Indigenous community members and healthcare providers. Six key informants including academics and experts on Indigenous health in south India were also interviewed (Table 2). Saturation of themes guided the data collection. A total of 24 instances of participant observation were carried out at the various villages and health facilities in Attapadi. Detailed fieldnotes were recorded and integrated into the analysis. The age of our participants ranged from 15 to 82 with both men and women from the Indigenous communities participating in the interviews and FGDs. These included parents of children studying in residential schools as well as male and female students who had come home for holidays.

**Table 2.** Sampling framework.

	*Muduga*	Kurumba	Irula	Total
Indigenous Community	IDI: 7, FGD: 1	IDI: 8, FGD: 4	IDI: 9, FGD:1	IDIs: 24, FGD: 6
Healthcare Providers	Doctors: 8 IDIs	CHWs^1^: 6 IDIs	Others^2^: 3 IDIs	IDIs: 17
Key Informants	Academia: 2 IDIs	Indigenous health experts: 4 IDIs		IDIs: 6
Participant Observation	6 instances	8 instances	10 instances	24 instances

^1^ Community Health Workers (CHWs) involved both Indigenous and non-Indigenous frontline healthcare workers working in the government health system in Attapadi

^2^ Staff working at the various health facilities other than doctors, nurses or CHWs

### Data analysis

An inductive approach to coding the transcripts was adopted to allow the various themes to emerge from the data (Miles et al., 2014). Once the initial open coding was complete, axial coding was used to express the relationships between the various themes as they arose from the data. This approach to analysis was taken to let the data drive the categories and themes rather than using a deductive or a quasi-deductive approach of fitting the findings to a particular framework. Coding of transcripts was carried out using the software package ATLAS.ti 8.4.2. ATLAS.ti 8.4.2 is a qualitative research tool used for coding and analysing data including transcripts, fieldnotes and photographs.

### Establishing trustworthiness and rigour

In order to ensure that our results were trustworthy and reflected the reality of the communities where we carried out research, multiple strategies including different forms of member checking (Harvey, 2015; Lincon & Guba, 1985) and triangulation were used to validate the data. Key findings were presented and discussed with all participants, with the opportunity to provide comments or add new data if relevant. The feedback that was obtained during this member checking process was also integrated into findings presented in this article. Data source triangulation (Patton, 1999) was carried out by comparing the perspectives of different stakeholders on the key findings. Methodological triangulation (Patton, 1999) was carried out by comparing the data that was generated across IDI, FGDs, and participant observation.

### Ethics approvals

Informed consent was gained from individual participants prior to data collection, and appropriate cultural and community permissions gained as discussed above. The Human Research Ethics Committees of the University of Canberra (20180074) and the Indian Institute of Public Health Delhi (IIPHD_IEC_03_2018) provided ethical approval. Regulatory permissions were obtained from the Kerala Department of Health (GO(Rt)No2677/2018/H&FWD), as well as the local administration in Attapadi.

## Findings

### Fear about losing Indigenous identity

Several participants, especially those who were village chiefs and older members of the Indigenous communities, argued that the practice of Indigenous children studying in residential schools, away from their villages, had a detrimental effect when it came to their ability to learn the skills that were expected of the young Indigenous in their communities. Several village chiefs noted that respect for the land and their unique agricultural practices were integral to “being an Indigenous” person. However, given the reliance on oral traditions for the transmission of skills and knowledge from one generation to the next, being away from the community meant that young people lost opportunities to observe and learn important skills and knowledge from their elders that were considered essential to their identity as an Indigenous person in the community. These skills included agricultural skills, knowledge of the forest and its ecosystem, knowledge of Indigenous healing traditions and the ability to identify herbs to treat different ailments:

You tell me who is an Indigenous person? … Is it because it says so on their birth certificate? Being an Indigenous person is far more than merely having it mentioned in a document. Our younger generation all have it mentioned in their document, but do they know our way of life, do they value it, I don’t think so. The future is bleak for us precisely because of that. (Indigenous community, IDI, male, TK 4)

One of the village chiefs described how he was unsure if his son could take over the task of being a village chief after his lifetime as he was studying in a residential school for most of the time:

Traditionally, my son must take over the responsibility of being the chief of our village once my time is over. But how can he be a village chief? He knows nothing about our way of life. If he was here with me, he would observe and learn from me. That is how I learnt from my father. But that opportunity is not there for our younger generation. (Indigenous community, IDI, male, TK 12)

Another area where loss of Indigenous heritage was discussed was Indigenous languages and religious rituals. Several Indigenous participants across the three different Indigenous communities spoke of how their native languages were dying, as their children were taught in Malayalam (the official language of the state of Kerala) at school. As they were away from their villages during important days when rituals were performed, participants argued that their children did not know the significance of such rituals and were at a loss when they returned to the villages after completing their education:

I used to call my mother *avva*, my daughter calls me *amma*. You might think there is nothing so important in this. But many of our songs, our culture is communicated through our language. So, it is a loss. (Indigenous community IDI, female, TM4)

The most important festival that is related to our identity here is the *Malleswaran* festival. Our children cannot attend it as they are away in their boarding school. They don’t see or participate in all that we do as a community during those days. Neither the rituals nor our dances, nothing. Then what culture are you saying they are going to learn? (Indigenous community, FGD, TK 3)

### Shame about Indigeneity

Many of the young Indigenous people interviewed spoke about their experiences of being stereotyped and labelled as an Indigenous person (*Adivasi*) during their time away in residential schools, and how this impacted their psychological wellbeing. They spoke of how non-Indigenous people referred to them using terms that signified they were lower in the social hierarchy. One young Indigenous participant who spoke about his experience said that he felt that the very term that is used to refer to Indigenous communities in official records is demeaning:

I hate that term “*pattigajathi*”. We are always referred to by that term and it is as if we are lower than others. Every time I hear that term, I feel so angry. (Indigenous community, IDI, female, TI 12)

On exploring this issue with other participants, it emerged that was a commonly held belief that the perception of Indigenous communities as lower in social hierarchies was one of the reasons why young Indigenous people felt awkward in identifying themselves as Indigenous:

There is a sense of shame among the youngsters here in identifying themselves as Indigenous. You should go and check their social media profiles, especially Facebook. They will have *Palakkad*, *Mannarcaud* and all sorts of places listed as their home– not *Attapadi*. Why? Because *Attapadi* means you are an *Adivasi*. And they don’t want the world to identify themselves as an *Adivasi*. (Healthcare provider, IDI, male, MO 4)

Several participants across different communities felt that the perception of Indigenous communities as “uncivilised savages” persisted among the non-Indigenous Indian population. They argued that this bias coloured the way in which others viewed them, including those involved in the formal health and education systems:

My neighbour who is non-Indigenous had some guests from another part of Kerala. They wanted to meet someone who is *Adivasi*. So, he brought them to my home. When they saw me, they were surprised, I was wearing shirt and trousers just like them. Probably they had this image that *Adivasis* are roaming in the forests with bows and arrows. [Laughs] they went back disappointed on seeing me. Even in 2018, this is unfortunately true. (Indigenous community, FGD, TM 5)

### Change in the attitudes of children when they returned

Many of the older Indigenous participants, including village chiefs, described young Indigenous children as showing a marked disregard for their customs and traditions when returning from residential schools. Participants noted that after spending time at the schools, young Indigenous children were reluctant to learn the lifestyle and customs of their community. Young people returning home to the community during holidays appeared to be uncomfortable being in the villages and tended to spend most of their time away at larger town centres:

Something changes in our kids. I can’t pinpoint it exactly, but it changes. I have seen it in both my children and other young people in my village. You know that change in attitude as if we are all not good, as if our ways and traditions are all backward. And that shows in the lack of interest in staying here or learning anything from us. (Indigenous community, IDI, male, TK 3)

The lack of integration of young Indigenous people who had studied in residential schools and subsequently returned to Attapadi was also highlighted by officials working in the Integrated Tribal Development Programme (ITDP) in Attapadi. They identified it as a cause of concern, as a good proportion of Indigenous youth were not taking up employment or pursuing any further studies after finishing their schooling. The ITDP officials pointed out that a key purpose of ensuring free education through the residential schools was to improve educational, workplace and social outcomes for Indigenous children:

If you go to *Agali* town centre, you will see groups of youngsters, mostly *Adivasi* youth. They have studied sometimes up to 10th, sometimes up to 12th [year of schooling]. But they spend most of the time there on their bikes with friends. Neither are they contributing in their homes nor are they taking up some gainful employment. (Fieldnotes, September 2018)

Study participants included Indigenous young people who had obtained government jobs but resigned after a short period of employment and returned to their villages. Discrimination and the inability to conform to expectations were cited as key reasons for discontinuing in those positions:

I left because they used to see us tribal staff differently from the others. I did not like it, so I quit my job. (Indigenous community, IDI, female, TM 8)

I could not fit into that lifestyle, so I left my job after six months and returned to my village. I don’t want to go back; I will take care of my cows and live here only. (Indigenous community, IDI, male, TI 13)

### The feeling of being neither here nor there

A key issue that emerged during interviews was the confusion that Indigenous young people felt on returning to their villages post their education in residential schools. After living away for several years they found aspects of life in their communities were alien to them when they returned. As customs and traditions of these communities are primarily transmitted through oral tradition and experiential learning, Indigenous youth who returned after several years were unable to relate to many of these traditions. Many skills that are essential to live the Indigenous way of life were lost to them, such as the ability to identify herbs and nutritious plants, the ability to predict the coming season and the kind of crops that had to be sowed. At a deeper level, some of the village chiefs and elders felt that the Indigenous philosophy of life was in conflict with what was held up as ideal for their youngsters. They felt this was a grave threat to preserving their way of life:

How will my son understand our way of life or live in our village. Once our young people are educated, they don’t want to live here. They want to go to the city and make more money. At the end of it are they happy? I don’t know that. There is a big difference in our thinking on these issues. So, to be successful in the way it is defined by the state and others, our young people need to leave our villages and live like you. They cannot really be “Indigenous” as that is not successful in the eyes of the state and others. (Indigenous community, IDI, male, TK 4)

Young Indigenous participants also reported that when they returned to their village after residential schooling, they felt they were neither fully accepted in non-Indigenous communities, nor were they able to live in an integrated manner in their villages. This led to a loss of identity and psychological distress:

People outside will never accept us fully; they won’t say it to our face, but it is apparent in their behaviour. The moment they know I am an Adivasi from Attapadi, then their attitude changes. So even after all these years, we cannot really be a part of them. (Indigenous community, IDI, female, TI 12)

### Stress due to the difficulty to fit in

One of the Indigenous participants with several years of experience working on Indigenous health issues in Attapadi explained that he felt the years of being away from their communities, its effect on being unable to integrate fully back into their community, and the associated loss of identity was a cause of stress and anxiety for young Indigenous people. When probed further on the issue, he spoke of how young people who had mostly studied in the schools located in Attapadi or nearby and were able to visit Attapadi often were far less anxious when it came to participating in the cultural life of the community, as opposed to those who lived in distance residential schools and who were not able to take part in the cultural life of the community until they finished their schooling. However, in his opinion, this was not even recognised as children going to boarding schools and staying there for a major portion of their growing years was considered the best practice:

When you go to the villages, you can make out the difference between those of our children who have grown up mostly in Attapadi and those who have not. The ones who have spent most of their time away are wary of others and will hardly interact with you. (Indigenous community, IDI, male, TM 8)

This was reiterated by several village chiefs and elders who pointed out that those young people who returned from residential schools found it far mor challenging to integrate into the life of the community:

Something changes, I have seen this happening to our young people when they return from schools. They find it difficult to live here or integrate themselves into their families or community. It is not just about knowing skills; I think something changes in their minds. It is like being here is so difficult for them. (Indigenous community, IDI, male, TK 3)

## Discussion

There is extensive evidence from countries around the world that the historical practice of forcibly removing Indigenous children from their communities and placing them in residential schools aligned with the dominant culture has substantial and ongoing negative physical and psychological impacts on both the youth themselves and their wider communities. These impacts include exposure to abuse, intergenerational trauma, mental distress, poorer physical health and the loss of languages and culture (Australian Institute of Health and Welfare, 2018; Menzies, 2010; O’Neill et al., 2018; Wilk et al., 2017). Clarity on collective or cultural identity has been shown to be important in the self-esteem and wellbeing of both individuals and communities, especially those who have experienced intergenerational trauma due to colonisation such as Indigenous communities (Taylor & Usborne, 2010). In this study of the current use of residential schools for Indigenous youth in the south Indian region of Attapadi we identified similar concerns and outcomes. Indigenous community members reported that, due to the physical relocation of Indigenous children away from their communities and families, traditional knowledge systems such as oral traditions and learning from the land were being disrupted. This led to young Indigenous people lacking cultural knowledge and skills that are considered necessary to live in their traditional lands. Indigenous elders identified gaps in cultural knowledge and experiences by these young people that are salient features of Indigenous identities. Community elders also expressed concern at the change in attitude that they observed among those who attended residential schools, including an acquired devaluing of the Indigenous way of life. The elders cited these issues as serious threats to the long-term cultural sustainability of their communities.

Our findings are consistent with recent work among Indigenous communities in the same geographical region which examined current pedagogies at residential schools, and found that both the content and approach were disconnected from what the Indigenous communities desired for their children and alienated them from their culture and traditions (Maithreyi et al., 2022). Several reports also identified high suicide rates and dropouts of Indigenous young people from their schools in Kerala and other parts of India despite various measures that have been taken for their wellbeing (Kuruvilla, 2022; Prasanna, 2017; Premkumar, 2020). A key factor strongly associated with the mental health and wellbeing of children and young people in multiple settings is resilience (Mesman et al., 2021). Among Indigenous communities, vital elements that have been shown to promote resilience among young people include a strong cultural identity (Andersson & Ledogar, 2008; Sasakamoose et al., 2016; Young et al., 2017), connection to the land (Hansen & Antsanen, 2016; Usher et al., 2021), community (Lalonde, 2006; Usher et al., 2021) and ancestors (Liebenberg et al., 2015; Toombs et al., 2016). A strong cultural identity has also shown to play an important role in preventing suicide among Indigenous communities (Pollock et al., 2018). The current system of residential schools in India appears to disconnect Indigenous young people from these factors, thereby making them more vulnerable to poor mental health and negative impacts on their overall wellbeing. Separation from caregivers and broader family members also threatens attachment relationships and creates potential trauma. Absence from their communities and exposure to influences alienating them from their culture and traditions also makes re-integration into communities difficult for Indigenous youth upon their return. Hence, it would be important to ensure that schools and other educational institutions for Indigenous communities are established as close as possible to the communities. This will facilitate greater connections to their traditional land, reduce the disconnection observed from cultural practices and beliefs, and reduce the period of absence of Indigenous children from their families and communities. The inability of young Indigenous people in India to thrive in the current educational system is not by accident but rather by design and a remanent of colonial and structural racism that continues to privilege dominant communities, their world views, and ways of life.

Formal education provides pathways to economic opportunities and wellbeing. However, an inherent feature of the current system of education for Indigenous youth in Attapadi and elsewhere in India is the promotion of deficit discourses regarding Indigenous communities. A deficit discourse frames and represents Indigenous communities, their culture, and traditions in a narrative of negativity, deficiency, and failure (Fogarty et al., 2018). Key forms in which this manifests includes the privileging of non-Indigenous languages and pedagogy and the promotion of stereotypes about Indigenous peoples in the syllabus and throughout schooling experiences. The current research suggests that exposure to this discourse within schools leads to the devaluing of Indigenous culture and traditions among Indigenous youth who attend these schools. It also leads to “othering” and the feeling among young Indigenous people that they would never be accepted as equals by others. Coupled with the struggles experienced by affected young people to integrate themselves back into their communities, Indigenous youth in this study reported feeling like they did not really belong anywhere. Research conducted among other Indigenous communities in India suggests that these experiences are shared by Indigenous youth across India (Gupta & Padel, 2020; Padel & Gupta, 2021; Sundar, 2010).

Our work highlights potential implications for the design and delivery of mental health services among Indigenous communities in India, especially for young people. Cultural continuity, rather than assimilation, has been shown to be an important protective factor when it comes to health and wellbeing of Indigenous communities (Chandler & Lalonde, 1998). The current residential school system interrupts this continuity, engendering vulnerability to mental health conditions including depression, anxiety, and suicide among young Indigenous people. Research among Indigenous communities in other parts of India has shown that interruptions to cultural continuity have a negative impact on mental health (Devarapalli et al., 2020). However, proposed solutions for addressing the mental health of Indigenous communities to date have included explicit emphasis on further integration into dominant cultures rather than ensuring cultural continuity (R. C. Mishra, 2015). That this approach is still being advocated is of critical importance given the criticism that the approach to mental health service provision for Indigenous communities in India, including young people, has focused on individualised treatments, and neglected important cultural and social issues (Kottai, 2018). This is in keeping with the process by which dominant cultures mediate not only how mental health and wellbeing is perceived, but also the kinds of solutions that are designed and implemented to improve mental health (Kirmayer, 2012).

Awareness of intergenerational trauma and its impact on mental health not only will help to address individual level experiences but also promote community level healing (Bombay et al., 2014). Efforts to promote mental health services in Attapadi and elsewhere for Indigenous communities have missed this crucial aspect and focused on institution-based, individualised biomedical treatment regimens that are culturally unsafe (Kottai, 2018; Shaji, 2019; Thomas, 2019). Work carried out in multiple contexts has shown how mental health providers need to reflect on the critical role that culture and intergenerational trauma play in mental health and wellbeing (Dagsvold et al., 2020; Kleinman & Benson, 2006). The failure to do so not only influences the quality of care that is provided but also stigmatises entire communities. Addressing this will require sensitising and training—mental healthcare providers will need to reflect on their understanding of Indigenous culture and its interaction with dominant cultures as well as the role of structural determinants that promote poor mental health outcomes. It is also important that Indigenous communities are engaged in the design and delivery of mental health services rather than a top-down approach involving non-Indigenous experts. Work carried out among Indigenous communities in other contexts has shown that efforts to improve mental health among Indigenous communities are most effective when communities are actively involved (Nelson & Wilson, 2017).

While the current research reveals key insights into the impact of residential schooling on Indigenous youth and their communities in Attapadi, it is important to note the diversity of Indigenous communities across India. Future research is required to further investigate whether these impacts are similarly reported in other Indigenous communities and contexts in India, where there may be a range of other contributing or mitigating factors. Additional focus on the impact on familial and caregiver-level relationships is also required. Future work in this area should also explore in greater depth the impacts of the current approach to education on mental health and resilience at the individual and community levels, including both qualitative and quantitative measures, as well as map pathways to improving the resilience of Indigenous young people and identify ways of promoting education that are culturally safe and facilitate greater wellbeing of Indigenous communities in India. The impact of other factors that influence the educational experience for both Indigenous children and their families including school distances, language of instruction, quality of teachers, and the active as opposed to symbolic participation of Indigenous parents in decision-making bodies of schools should also be investigated to understand how they impact on the mental health and overall wellbeing of Indigenous children in India.

## Conclusion

The assimilatory approach to the education of young Indigenous people in India mirrors the overall approach in Indian policy, namely that it locates the causes of poor health and development of Indigenous communities in their culture and traditions (Xaxa, 2012). According to this view, removing children from their communities and ensuring that they are taught the culture, language and customs of the dominant community reflects the efforts of the state to ensure improved outcomes for Indigenous youth. However, the current research and wider evidence from other countries suggests this approach has a myriad of negative impacts and long-term harms on the wellbeing of Indigenous young people and their communities. Addressing this requires a more detailed understanding of the specific factors influencing outcomes for Indigenous youth within the Indian residential schooling system, and designing and implementing data-informed structural, policy, and conceptual change in the education of Indigenous youth in India.

## Notes

1. While Adivasi or Scheduled Tribes are the commonly used terms in India to denote those belonging to the Indigenous communities, we chose to use the term Indigenous in this paper. The Indigenous category focuses attention on the larger social and political determinants of wellbeing such as the continuing colonisation and expropriation of tribal lands, forests, and other resources by dominant groups.

2. Ashrama Shala is an ancient practice in India where children primarily belonging to the Brahmin and Kshatriya Hindu communities leave their homes and live with a teacher.

3. The Tribal Sub-Plan was an important policy measure aimed at the rapid socioeconomic development of Indigenous population in India. It formed a part of annual plan of State/Union Territory to give specific benefits to Indigenous communities.
